# Quality of Life in Amyotrophic Lateral Sclerosis Patients and Care Burden of Caregivers in Sardinia during COVID-19 Pandemic

**DOI:** 10.3390/healthcare11111641

**Published:** 2023-06-03

**Authors:** Davide Gentili, Giovanna Deiana, Vanna Chessa, Annalisa Calabretta, Elisabetta Marras, Costanzo Solinas, Carmelo Gugliotta, Antonio Azara

**Affiliations:** 1 Public Health Office, Local Health Unit 2 Marca Trevigiana, 31100 Treviso, Italy; 2 Department of Biomedical Sciences, University of Sassari, 07100 Sassari, Italy; 3 University Hospital of Sassari, 07100 Sassari, Italy; 4 Home Care Unit, Ventilated Patients with High Care Complexity, Health District of Sassari, 07100 Sassari, Italy; 5 Health District II, ASL Roma 1, 00185 Rome, Italy; 6 Department of Medicine, Surgery and Pharmacy, University of Sassari, 07100 Sassari, Italy

**Keywords:** Amyotrophic Lateral Sclerosis, Quality of Life, caregivers, COVID-19

## Abstract

Amyotrophic Lateral Sclerosis (ALS) is a rare neurogenerative disorder whose median survival ranges from 2 to 4 years after symptomatic onset. Therefore, the global Quality of Life (QoL) assessment in these patients should be carefully evaluated to guarantee an adequate care level, particularly during the COVID-19 pandemic period, given the increased social isolation and the pressure on healthcare services. Caregiving has been recognized as an important source of physical and psychological burden, with a possible QoL impairment. The purpose of this study was to evaluate the QoL of ALS patients and the burden of their caregivers across Sardinia, Italy. The ALS Specific QoL Instrument-Short Form (ALSSQOL-SF) and the Zarit Burden Inventory (ZBI) tools were used to assess patient’s QoL and the burden on their caregivers, respectively. The questionnaires were supplemented with items specific for the COVID-19 period. Sixty-six family units of patients with advanced ALS were interviewed between June and August 2021 across Sardinia. Patients’ psychological and social well-being were found to significantly affect the patients’ QoL, regardless of their physical condition. In addition, the caregiver burden resulted as being inversely proportional to the patient’s perceived QoL. Lack of adequate psychological support was reported among the caregivers during the emergency period. Providing adequate psychological and social support might be useful to improve QoL in middle and late stages of ALS patients and to decrease caregivers’ perceived home care burden.

## 1. Introduction

Amyotrophic Lateral Sclerosis (ALS) is a progressive neurodegenerative disease that affects nerve cells in the brain and spinal cord, leading to muscle weakness, paralysis, and eventual respiratory failure. ALS can appear in different clinical conditions depending on disease phenotype, associated genetics, and neurophysiopathological mechanisms [1,2,3,4,5]. ALS’s estimated incidence in Europe is 1.75–3 cases per 100,000 people/year, with a prevalence of 10–12 cases per 100,000 people, although there are significant geographical differences [6,7,8]. Median survival ranges from 2 to 4 years after symptomatic onset and only 5–10% of subjects survive beyond 10 years [9,10].

ALS onset is very rare in youth and is mainly related to familial forms of the disease (fALS) [4]. Starting from the fourth decade of life, the incidence of ALS increases with age [11,12,13]. Juvenile-onset forms of ALS are most frequently found in isolated rural areas or islands, while cases with older onset are common in areas with high population density. This fact may be related to a founder effect in isolated regions or to the involvement of different environmental factors [6]. In the late stages of ALS, individuals experience significant physical, emotional, and psychological challenges that can profoundly impact their quality of life. These challenges include difficulty in communicating, mobility, feeding, and daily living activities, as well as increased risk of respiratory infections and other medical complications.

Therefore, Quality of Life (QoL) assessment plays a key role in degenerative diseases with poor prognosis, such as ALS. QoL has been defined by the World Health Organization (WHO) as an individual’s perception of their position in life in the context of the culture and value systems in which they live and in relation to their goals, expectations, standards, and concerns [14]. The current lack of effective treatments and the inexorable progression of this disease highlight the need to promote care models that can guarantee the best QoL to the patient, with a psycho-physical support suitable for the different stages of the illness. This is even more important during the COVID-19 pandemic period, given the increased social isolation and the pressure on healthcare services.

Despite these challenges, many individuals with late-stage ALS are able to maintain a meaningful QoL level through the support of their caregivers, adaptive equipment and technology, and palliative care interventions aimed at improving comfort and relieving symptoms. Caregivers are key figures who often play a key role in clinical decision-making [15,16,17]. Caring for a partner or family member with a progressive neurological disease has been recognized as an important source of physical and psychological burden, with a possible impairment in QoL [18,19,20,21,22,23]. Moreover, time spent for care activities and caregivers’ responsibilities generally increases with disease progression [24,25].

However, it is important to acknowledge the complex and often devastating impact of this disease on individuals and their families, and to prioritize compassionate and patient-centered care throughout the course of the illness. Therefore, the main purpose of this study was to evaluate the perceived QoL in ALS patients in Sardinia, Italy and the care burden of their caregivers across the region, through direct interviews at the patient’s house. 

## 2. Materials and Methods

### 2.1. Study Setting

**Figure 1:**
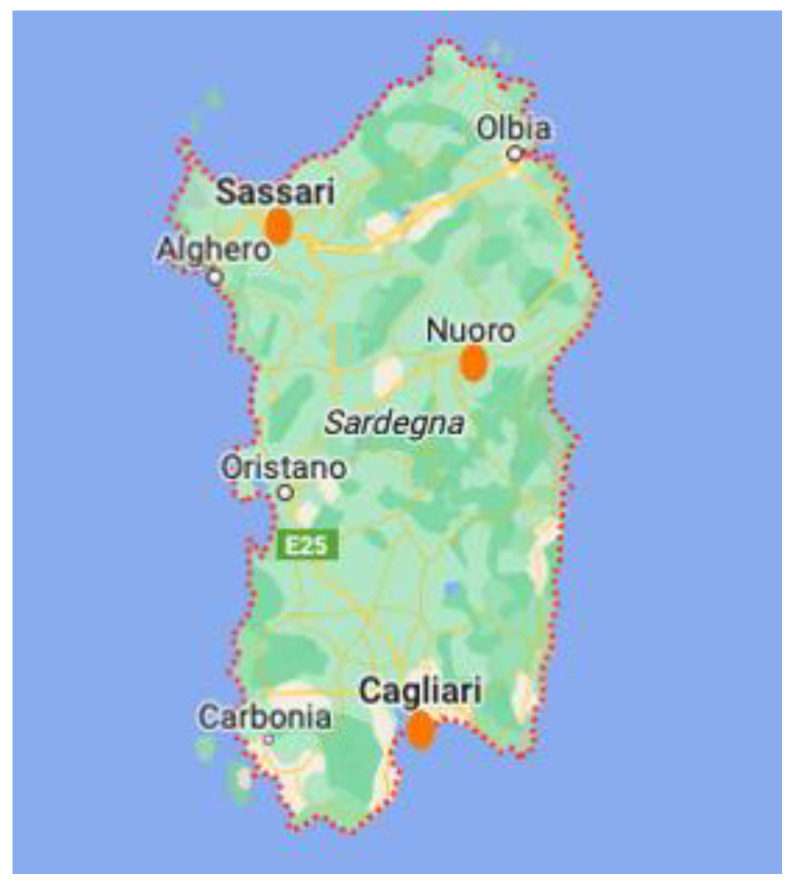
Sardinian Region with the three centers responsible for ALS diagnosis.

The study was conducted in Sardinia, an Italian region commonly divided into three zones (North, Center, and South) based on its oro-geographical characteristics and the presence of the main cities. Three main hospitals can be found on the island, where only three neurology units capable of diagnosing ALS are located: University Hospital in Sassari, St. Francis Hospital in Nuoro and “G. Brotzu” Hospital in Cagliari (Figure 1). After diagnosis, these patients are assisted at home through a project called “Returning Home” promoted by the Sardinian Region by a multidisciplinary team, consisting of an anesthesiologist, a nurse, and a psychologist [26].

### 2.2. Study Design

All data were collected through interviews conducted from June to August 2021 by the principal investigator, who personally visited at home each subject, in order to guarantee an homogeneous and repeatable method, with the support of the Italian Association of Amyotrophic Lateral Sclerosis (AISLA) and the help of dedicated multidisciplinary services across the Region (home and palliative care units). All subjects were recruited through these medical services.

All patients with a confirmed diagnosis of ALS in a late stage of the disease, classified as stage 4 according to the King’s clinical staging system, who required full time support from a caregiver, and medical assistance from specialized community services, were included in the study. Age (<40, 41–50, 51–60, 61–70, >80), sex, education, ethnicity, religious orientation, marital status, number of family members, date of diagnosis, presence of tracheostomy, respiratory support, and percutaneous endoscopic gastrostomy were recorded for each patient. Patients with Fronto-Temporal Dementia (FTD) and Completely Locked-In State (CLIS), verified during the patient’s home interview by clinical records, were excluded, and only their caregivers were interviewed.

For each caregiver, all of whom were family members, the following variables were recorded: age, sex, employment status, the interval between ALS’ diagnosis and the beginning of the home care activity, and the hours they dedicated to look after the family member (weekly total, estimated on a daily average, including weekends).

Before conducting the study, the investigator spent six months in a specialized multidisciplinary team unit that deals with patients with ALS, identifying the most relevant themes and issues. Questionnaires were administered after a qualitative interview with probe questions concerning appropriate communication of diagnosis, adequacy of hospital follow up, utility of the “Returning Home” regional project, support received in relation to bureaucratic aspects, adequacy of territorial home care services, supply of medical devices and medication, and training of home care personnel. Based on the answer provided, the interviewer determined the presence or absence of the required element, allowing for a percentage score to be formulated for each area of investigation. Caregiver and ALS patient questionnaires were conducted independently.

### 2.3. Questionnaire Design

A disease specific questionnaire that captures global QoL values is the ALS Specific Quality of Life instrument (ALSSQOL). This questionnaire, and its revised version ALSSQOL-R, have been designed by international ALS experts and validated in multicenter studies [27,28,29]. The ALSSQOL-R was also modified in a Short Form (ALSSQOL-SF), without significantly affecting the objectives of the original instrument [30]. Generic QoL questionnaires, such as the 36-item Short Form Health Survey (SF-36) [31] and the McGill Quality of Life Questionnaire [32], are not suitable for ALS patients, as they do not take into account specific aspects of the disease. The Sickness Impact Profile Assessment (SIP and SIP/ALS-19) [33] and the Amyotrophic Lateral Sclerosis Assessment Questionnaire (ALSAQ-40) [34] likewise are primarily based on physical function and fail to fully capture other key factors for global QoL assessment [35,36,37,38,39,40].

To interview ALS patients, the ALSSQOL-SF questionnaire was selected as a model for its versatility, handling easiness, reliability, and inclusion of the psychological, existential, spiritual, and support aspects. ALSSQOL-SF was modified (Section S1), adding two questions to examine the interaction of ALS patients with people and environment outside the family unit (items 21 and 22); in addition, eight questions were added to assess whether the COVID-19 emergency could have significantly affected patients’ QoL (QoL worsening, perceived loneliness, level of care, psychological support, lack of physical contacts, personal limitations, relationship with the caregiver, difficulties in communication).

The Zarit Burden Inventory (ZBI) is a tool that can be used to assess the consequences that long-term care of a family member with chronic degenerative diseases has on the caregiver. The items investigate how the patient’s disability impacts on the caregiver’s QoL, psychological suffering, guilt, financial difficulties and shame, generating social and family difficulties [41]. The ZBI has been validated in relation to three tools that evaluate different aspects of the person’s life and psychological–relational situation: General Health Questionnaire (GHQ-12), Hospital Anxiety and Depression Scale (HADS), Symptom Checklist 90 Revised (SCL90-R) [42].

The ZBI questionnaire was selected to interview caregivers because of its brevity, easiness of administration, and for the compliance with the GHQ-12, HADS, and SCL90-R tools. Seven additional items were identified to assess whether the COVID-19 emergency period could have affected the overall assessment. Scores were redistributed from 0 to 10 to improve the sensitivity of the rating scale, to make the data comparable among the two questionnaires, and to minimize the tendency of caregivers to exclude extreme responses (detected during the validation phase). In addition, to avoid the use of the words “never” and “always”, the value 0 has been represented by the term “Strongly disagree” and the value 10 by the term “Strongly agree”. Scores 1, 2, and 3 were expressed as “disagreeing” with the statement, 4 “partially disagreeing”, 5 “neither agree nor disagree”, 6 “partially agreeing”, 7, 8, and 9 “agreeing” with the statement (Section S2). The QoL value of 6 was set as the threshold for an acceptable quality of life. The multidimensional aspect of the ZBI in this population, considering factors such as social restriction, self-criticism, anger, and frustration, highlighted the ability of the tool to objectively focus on each caregiver’s specific aspect of burden in ALS home care and address intervention programs to support them [22,43].

The modified ALSSQOL-SF and ZBI questionnaires were validated (concept validation) by a team of medical doctors in public health with the support of a psychologist with several years of experience on ALS, after a preliminary evaluation of the tools among patients in the Ventilated Patient with High Care Complexity Unit in the district of Sassari. A double translation procedure of the original ALSSQOL-SF has been performed (i.e., two independent translations from the source language, and reconciliation by a third linguist) to ensure the equivalence of the source and a target version was obtained. In addition, the psychologist verified the semantic suitability.

### 2.4. Statistical Analysis

**Figure 2:**
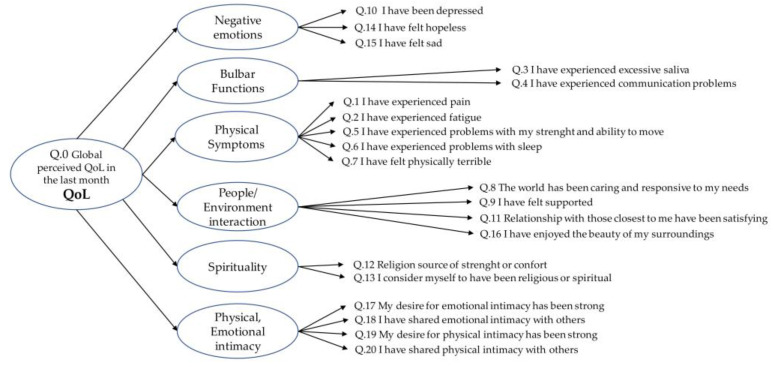
Item considered for each thematic domain, as reported in ALSSQOL-SF original form.

Qualitative data were analyzed quantitatively by the use of probe questions (calculated as the percentage of affirmative responses for each probe question, in the three analysis groups: North, Centre, and South Sardinia), while providing the insights of the qualitative information to fully understand its implications. Comparison of the questionnaire items among the different geographical areas in the Northern, Central, and Southern areas of the island, was carried out through a non-parametric analysis of variance (Kruskal-Wallis test). The items of the modified ALSSQOL-SF questionnaire were grouped into six thematic domains, as previously described [28,30]. The score of each domain was calculated for each patient as the mean of the specific items for that domain, as shown in Figure 2. Every domain has subsequently been related to the ALS perceived global QoL by using Spearman’s rho correlation. The value of each domain, for each patient, is given by the mean of the Likert scale 0–10 of the questions that make up each domain.

An ordinal logistic regression model was performed to assess the influence of the independent variables in the six thematic domains on the dependent variable Global QoL. The Ordinal Hosmer–Lemeshow test was used to evaluate the goodness of adaptation of the logistic regression model. Global scores of the modified ZBI between 0 and 52 were considered as “absent to mild burden of care”, between 53 and 102 as “mild to moderate”, between 103 and 152 as “moderate to severe”, and scores above 152 were defined as “severe to critical burden of care”.

Finally, the perceived QoL value among ALS patients was compared to the total score obtained from the ZBI questionnaire to probe whether there was a relationship between the two assessments. The common items of the questionnaires were compared using the Mann–Whitney U tests.

Microsoft Excel software (Microsoft Corporation, Redmond, DC, USA) (Professional Plus 2019, version 2302) was used for data entry, and IBM SPSS Statistics (Version 25.0. Armonk, NY, USA: IBM Corp.) was used for data analysis.

## 3. Results

### 3.1. Quality of Life of the ALS Patient in Sardinia—Modified ALSSQOL-SF

Sixty-six ALS subjects with an advanced stage of the disease were interviewed, equally distributed between North, Center, and South Sardinia. The mean age of the patients was 62.3 ± 10.7 years old. Males represented 67% of the subjects, mainly in the age group 51–70 years. The assessment of the typical presentation at disease onset was not possible for all interviewed patients. The time period elapsed from the diagnosis to the interview was 1825 days ± 1204 days. Forty-three subjects had a tracheostomy (65.2%) with invasive mechanical ventilation at the time of the investigation and 74.2% (n = 49) underwent gastrostomy. Fifteen subjects used a non-invasive ventilation device (NIV). Over 25% of the sample (n = 18) had CLIS or FTD at the time of the survey (Table 1).

The answers to the modified ALSSQOL-SF questionnaire in the 47 ALS subjects who did not have CLIS or FTD are reported in Table 2. Regarding the overall QoL perceived by the ALS subject in the last 30 days, the rating scale ranges from zero (terrible QoL) to ten (excellent QoL). The threshold value for considering the QoL acceptable is 6. For items 1–30, the rating scale is shown in the caption.

**Figure 3:**
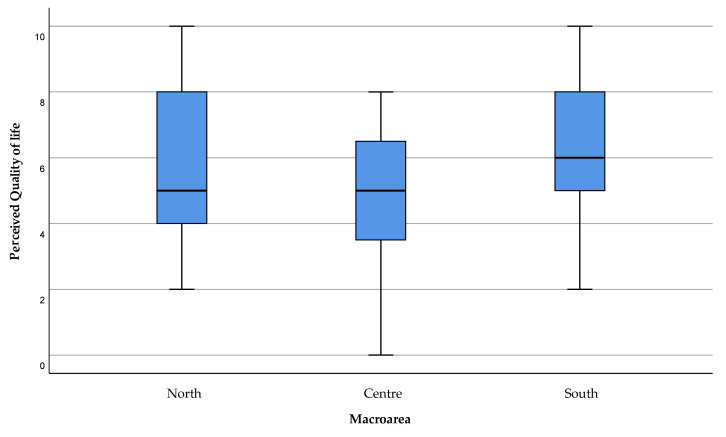
Overall quality of life perceived by the patient in the last 30 days. The value “0” stands for a “very bad” QoL, “10” for Excellent. The threshold for an acceptable QoL is “6”.

The answers to the modified ALSSQOL-SF questionnaire in the 47 ALS subjects interviewed, considered as a mean value of the 30 days prior to the interview, and during a period not affected by restrictions due to the COVID-19 emergency (1 June–31 August 2021), highlighted a value of QoL below the desirable threshold of acceptability for the areas of North (Sassari, Alghero, Tempio-Pausania, Ozieri) and Central Sardinia (Oristano, Nuoro), as shown in Figure 3. There is also greater variability in Northern Sardinia compared to the other areas of investigation. The Kruskal–Wallis test anyway does not detect a significant difference among the macro-areas (*p* = 0.290).

**Figure 4:**
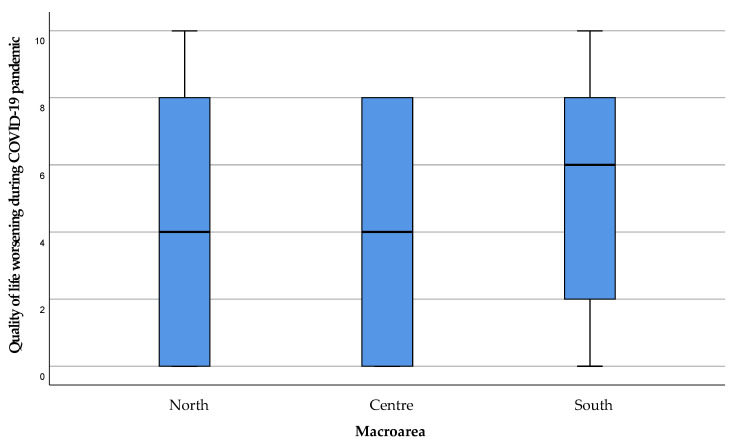
Perceived deterioration in the patient’s quality of life in the period of the COVID-19 emergency. The value 0 represented the expression “strongly disagree” with the statement, and 10 the expression “strongly agree”. Values 1, 2, and 3 were expressed as “disagreeing” with the statement, 4 “partially disagreeing”, 5 “neither agree nor disagree”, 6 “partially agreeing”, 7, 8, and 9 “agreeing” with the statement.

The influence of the COVID-19 period on the QoL of the subjects has been moderate in the areas of Northern and Central Sardinia (median 4), with a greater impact in the South of the island (Figure 4). A wide distribution of the response values can be seen in every area, excluding a significant difference between them (*p* = 0.987).

**Figure 5:**
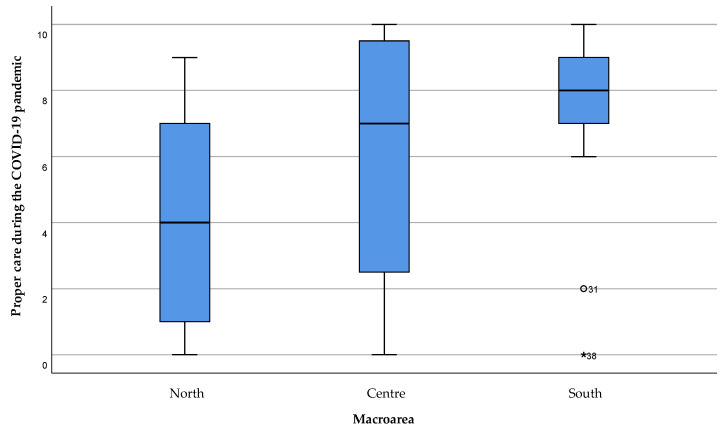
Patient perception of having received adequate care during the COVID-19 emergency. The value 0 represented the expression “strongly disagree” with the statement, and 10 the expression “strongly agree”. Values 1, 2, and 3 were expressed as “disagreeing” with the statement, 4 “partially disagreeing”, 5 “neither agree nor disagree”, 6 “partially agreeing”, 7, 8, and 9 “agreeing” with the statement. Extreme outliers are reported as *, mild outliers as °.

During the COVID-19 pandemic period, a significant difference (*p* = 0.023) between the patients’ perception of having received adequate care among the areas was observed, with a consistent worsening in Northern Sardinia compared to the areas of the South and the Center of the island (Figure 5). Moreover, a high variability can be seen for both Northern and Central areas.

**Figure 6:**
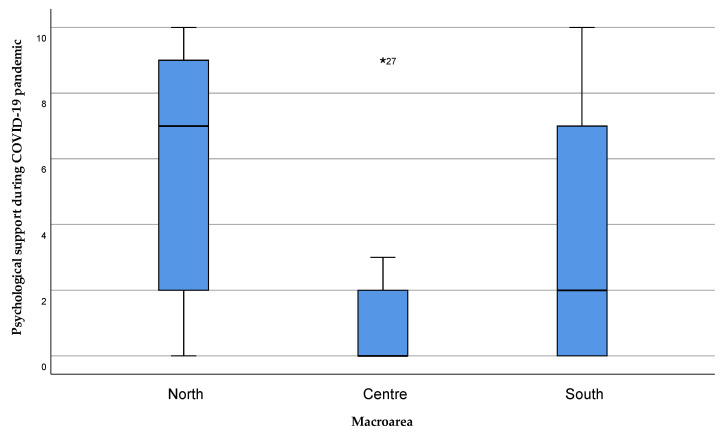
Perception of psychological support to the patient during the emergency. The value 0 represented the expression “strongly disagree” with the statement, and 10 the expression “strongly agree”. Values 1, 2, and 3 were expressed as “disagreeing” with the statement, 4 “partially disagreeing”, 5 “neither agree nor disagree”, 6 “partially agreeing”, 7, 8, and 9 “agreeing” with the statement. Extreme outliers are reported as *.

Psychological support was more satisfactory according to patients in Northern Sardinia, compared to the Central and Southern areas, with a statistically significant difference (Figure 6, *p* = 0.02). 

**Figure 7:**
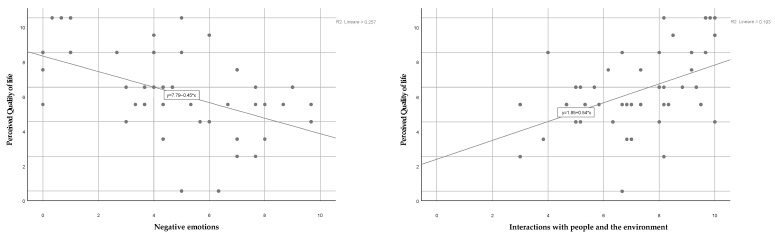

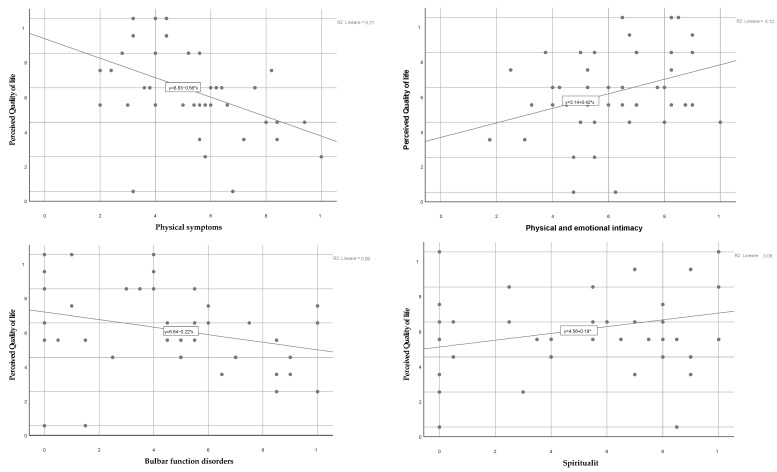
Relationship of the six domains investigated by the ALSSQOL-SF questionnaire (negative emotions, interactions with people and environment, physical symptoms, physical and emotional intimacy, alteration of bulbar function, spirituality) with the ALS patients’ perceived QoL value.

The six domains obtained from the ALSSQOL-R and ALSSQOL-SF questionnaires analysis methodology (negative emotions, interactions with people and environment, physical and emotional intimacy, spirituality, physical symptoms, alteration of bulbar function) were related to the QoL of the patients, as shown in Figure 7.

The QoL of ALS patients was positively related to the increase in domains such as interactions with people and environment, physical and emotional intimacy, and spirituality, whereas it was negatively conditioned by an inadequate bulbar function, physical symptoms and negative emotions. All the considered domains in the univariate analysis were significantly related to the global QoL variable (*p* < 0.05). An ordinal regression model (Table 3) was therefore carried out to assess the influence of independent variables on these domains on the global QoL dependent variable (Likert scale 0–10).

The proposed model highlighted that the independent variable “Negative emotions” was significantly associated with a decrease in the perceived QoL (odds ratio 0.70 versus the outcome satisfactory QoL, *p* = 0.006). The Ordinal Hosmer–Lemeshow test also showed an acceptable adaptation of the regression model (*p* = 0.2324).

### 3.2. Caregivers’ Burden—Modified Zarit Burden Interview

The main characteristics of caregivers for Northern, Central, and Southern Sardinia are reported in Table 4.

Results of the modified ZBI questionnaire are presented in Table 5. The rating scale of the ZBI is reported in the table caption.

The ZBI global score showed moderate to severe burden in the 38% of familiar caregivers, and critical burden in the 9% of the sample. Only 17% of the caregivers reported absent to mild burden, and 36% mild to moderate.

**Figure 8:**
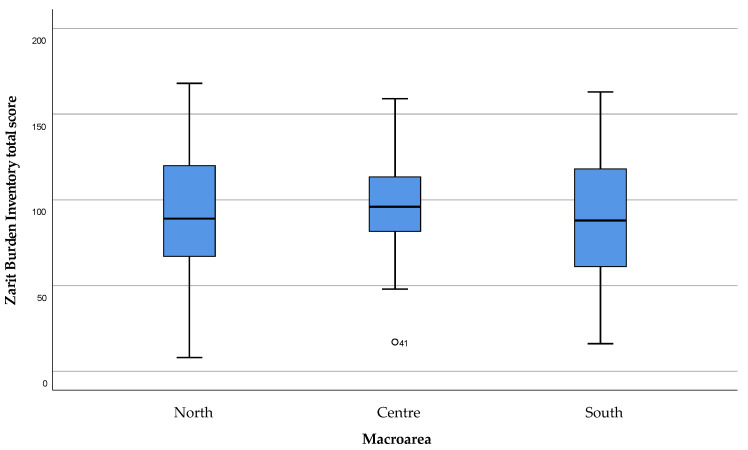
Caregivers’ Perceived Care Load. Modified ZBI scores in the range 0–52 were considered as “absent to mild burden of care”, 53–102 as “mild to moderate”, 103–152 as “moderate to severe”, and above 152 as “severe to critical burden of care”. Mild outliers as **°**.

Global perceived care burden, obtained from the ZBI total score, showed a similar value for moderate care load in each area of the island (Figure 8).

**Figure 9:**
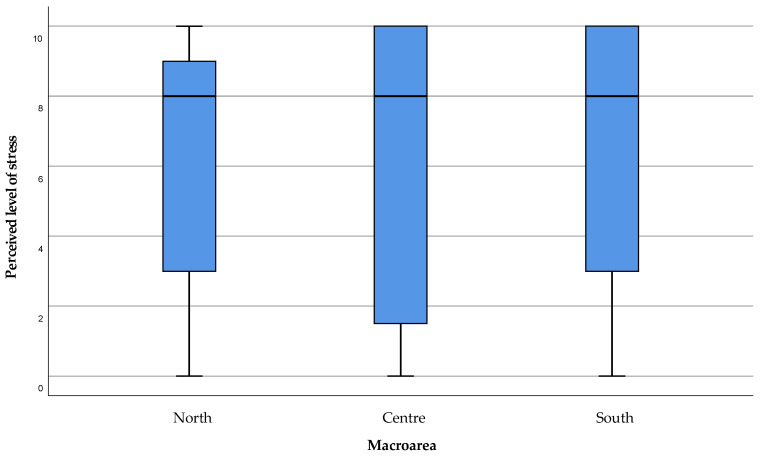
Level of stress perceived by caregivers. Scores in the range 0–4 were considered as “absent to mild level of stress”, 5–6 as “mild to moderate”, 7–8 as “moderate to severe” and above 8 as “severe to critical level of stress”.

**Figure 10:**
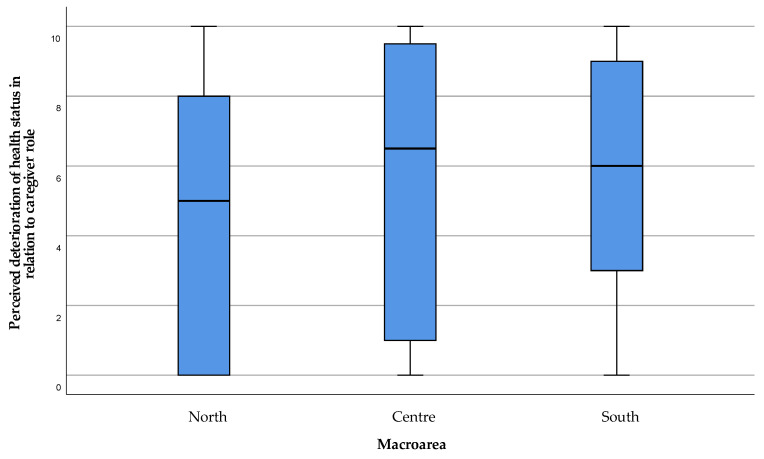
Perception of the decrease in the state of health of caregivers in relation to the care activity provided. The value 0 represented the expression “strongly disagree” with the statement, and 10 the expression “strongly agree”. Values 1, 2, and 3 were expressed as “disagreeing” with the statement, 4 “partially disagreeing”, 5 “neither agree nor disagree”, 6 “partially agreeing”, 7, 8, and 9 “agreeing” with the statement.

Although the care burden seems to be moderate, the stress level perceived by the caregiver in caring for the family member as well as dealing with other family members and work responsibilities (item 3) highlights a high level of perceived stress in all the areas of the island (Figure 9). This issue is also expressed by the level of personal health status perceived by the caregivers (item 10), which shows a potential hazard for their health state in connection with the continuous care activity provided (Figure 10).

**Figure 11:**
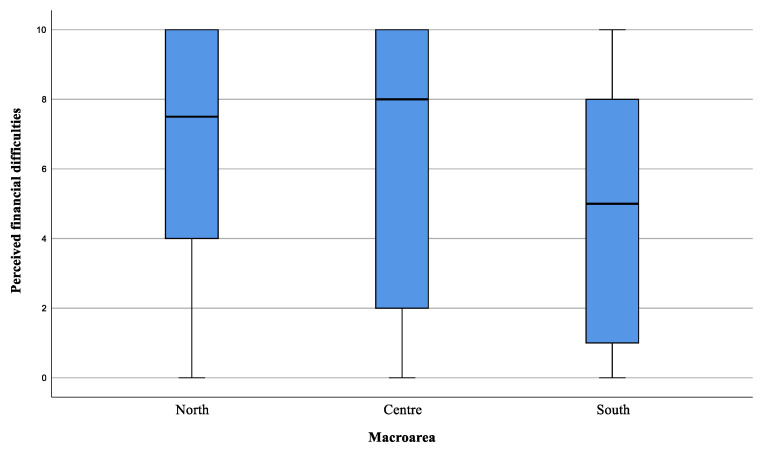
Economic difficulty perceived by caregivers, in addition to personal expenses, to cope with care. The value 0 represented the expression “strongly disagree” with the statement, and 10 the expression “strongly agree”. Values 1, 2, and 3 were expressed as “disagreeing” with the statement, 4 “partially disagreeing”, 5 “neither agree nor disagree”, 6 “partially agreeing”, 7, 8, and 9 “agreeing” with the statement.

Moreover, the assessment of the caregivers’ economic burden (item 15) showed a possible financial difficulty in meeting all the expenses related to care, especially in the North and Centre of the island (Figure 11).

**Figure 12:**
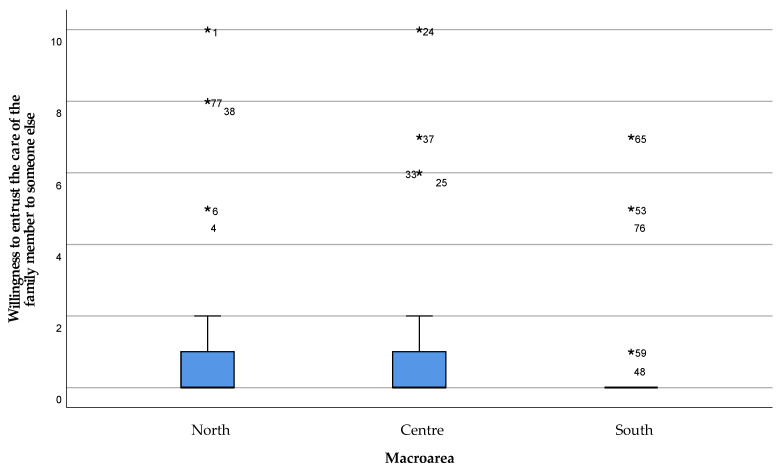
Willingness to entrust the care of the family member to other people. The value 0 represented the expression “strongly disagree” with the statement, and 10 the expression “strongly agree”. Values 1, 2, and 3 were expressed as “disagreeing” with the statement, 4 “partially disagreeing”, 5 “neither agree nor disagree”, 6 “partially agreeing”, 7, 8, and 9 “agreeing” with the statement. Extreme outliers are reported as *.

Nevertheless, the caregivers strongly disagreed to the chance of entrusting the patient’s care to people outside the family, with a median response value of zero (strongly disagree) and the upper extreme not an outlier in the boxplot that does not exceed the value of 2 (disagreement) (Figure 12).

**Figure 13:**
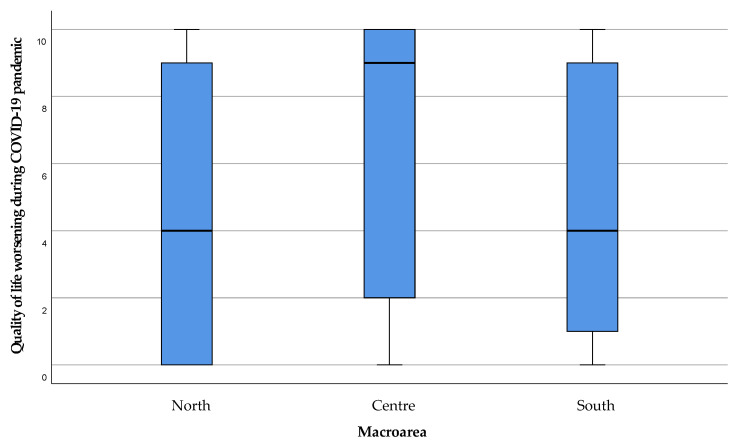
Perceived deterioration in caregiver quality of life during the COVID-19 period. The value 0 represented the expression “strongly disagree” with the statement, and 10 the expression “strongly agree”. Values 1, 2, and 3 were expressed as “disagreeing” with the statement, 4 “partially disagreeing”, 5 “neither agree nor disagree”, 6 “partially agreeing”, 7, 8, and 9 “agreeing” with the statement.

The assessment of the caregivers’ QoL worsening during the COVID-19 emergency period showed a mild worsening of QoL in the North and South of Sardinia, with a major decline in the Center, but no significant difference (Figure 13).

**Figure 14:**
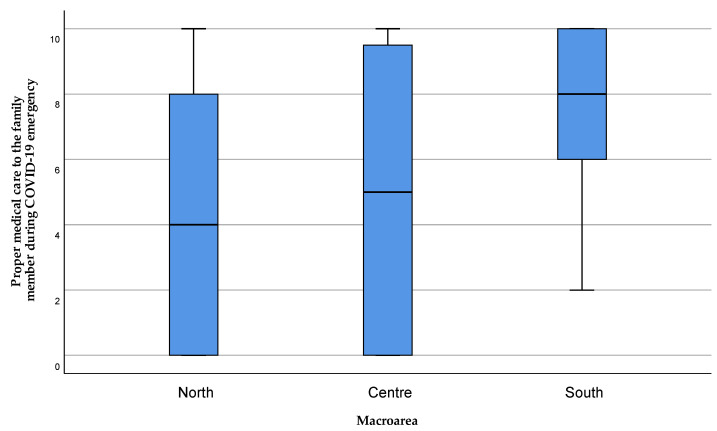
Caregivers’ perception of the care received by family members during the COVID-19 emergency period. The value 0 represented the expression “strongly disagree” with the statement, and 10 the expression “strongly agree”. Values 1, 2, and 3 were expressed as “disagreeing” with the statement, 4 “partially disagreeing”, 5 “neither agree nor disagree”, 6 “partially agreeing”, 7, 8, and 9 “agreeing” with the statement.

Instead, the caregivers’ perception of their family members having received adequate care during the COVID-19 emergency showed a significant difference (*p* = 0.008) between the areas of Northern and Central Sardinia compared to the South (Figure 14).

**Figure 15:**
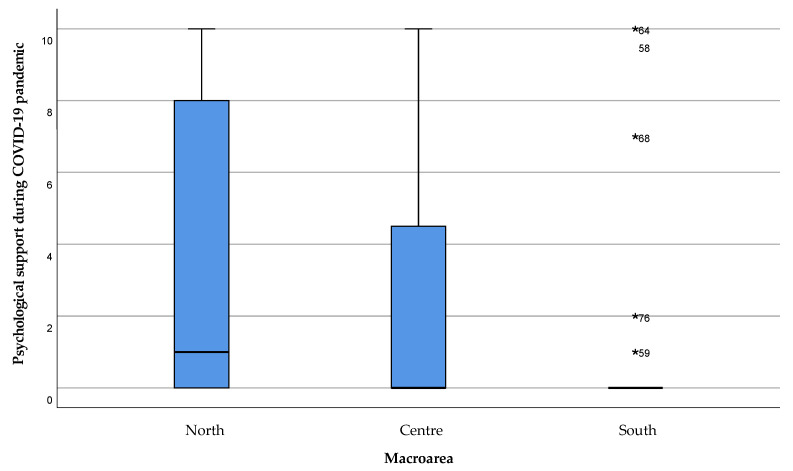
Perception of psychological support received by caregivers during the COVID-19 emergency. The value 0 represented the expression “strongly disagree” with the statement, and 10 the expression “strongly agree”. Values 1, 2, and 3 were expressed as “disagreeing” with the statement, 4 “partially disagreeing”, 5 “neither agree nor disagree”, 6 “partially agreeing”, 7, 8, and 9 “agreeing” with the statement. Extreme outliers are reported as *.

Finally, the psychological support provided to the caregivers during the COVID-19 emergency was higher in the areas of Northern Sardinia, especially in the Sassari area, compared to Central and Southern Sardinia, with a statistically significant difference (*p* = 0.047). Nevertheless, the median value in every area highlights the lack of adequate psychological support to the caregiver during the emergency period (Figure 15).

**Figure 16:**
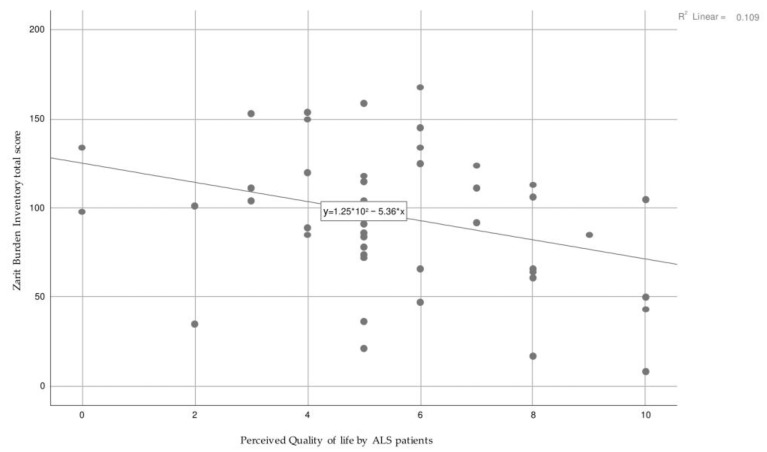
Relationship between the caregiver’s care burden and the Quality of Life perceived by the ALS subject.

The direct comparison between the care burden perceived by the caregiver and the QoL of the ALS subject (Figure 16) showed a statistically significant correlation (*p* = 0.02). In particular, as the total perceived care burden increases, there is a decrease in the QoL of the ALS subject.

The direct comparison between ALS patients and caregivers for the common questionnaire (Mann–Whitney U tests) items showed a statistically significant difference (*p* = 0.029) in the perception of the psychological support received during the emergency (item 26), with insufficient support among the caregivers group.

**Figure 17:**
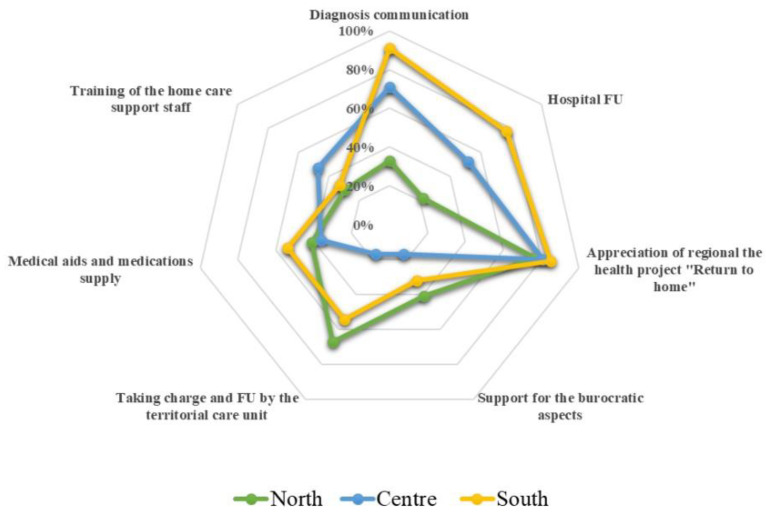
Satisfaction rates with respect to the topics detected with the probe questions during the qualitative interview.

The results of the probe questions carried out during the qualitative part of the interview (Figure 17) highlight potential difficulties in the areas of training of the home care support staff, medical aids, and medication supply, and support for the bureaucratic issues. Moreover, the interview reveals that the diagnosis communication, the patient’s taking charge, and his follow up by the territorial care unit vary significantly between the North and the South of the island.

## 4. Discussion

The periodic evaluation of the QoL among ALS subjects and their caregivers is fundamental to identify the possible critical issues and fix them. The knowledge of the factors affecting QoL allows one to have a clinical management that is not only limited to the survival of patients, but that has the primary objective of humanizing care and improving the QoL of the subject himself. The achievement of this objective requires the availability of an assistance service that guarantees a psycho-physical continuity of care after the hospital diagnosis. Psychological assistance and social support should therefore be integrated into the multidisciplinary team in the territory, which must meet all the needs expressed in the different phases of the disease, ensuring in a reasonable time interval to be able to provide a service at the patient’s house, or in the hospital if the service is not otherwise possible at home.

A decrease in QoL perceived by the subject is not necessarily related to the disease progression or to a reduction in the physical function. Psychological health is an important part within the QoL assessment sphere and does not necessarily decrease when physical strength and autonomy are lost [39,44,45,46,47]. The QoL observed in our study sample was found to be below the threshold value of suitability for the areas of Northern and Central Sardinia, reaching an acceptable median value only in the South. A possible explanation is that the territorial organization of services and the higher presence of care facilities and personnel in the Southern part of the island may have ensured a more satisfactory level of care. In the North and Center, especially during the COVID-19 period, care resources were allocated for pandemic management. High scores in the domains related to interactions with people and environment, physical and emotional intimacy, and spirituality have shown a possible positive effect on the QoL [48,49,50]. Conversely, the presence of issues in bulbar function, physical symptoms, and negative emotions showed a possible negative impact on QoL [51,52,53,54,55]. Negative emotions resulted in a statistically significant predicting factor of QoL decrease in a subsequent ordinal logistic regression analysis.

Conversely, a strong family support has been associated with better QoL for patients, greater life satisfaction, increased self-esteem, and better coping abilities with the disease [11,56,57]. This means that interventions aimed at improving the quality of family interactions can have a positive impact on the lives of ALS patients, but family caregivers must be adequately supported [58,59].

The COVID-19 period seems to have had a greater influence on the perceived QoL of the ALS subjects in southern Sardinia than in the Northern and Central areas. The main issue in the North may have concerned the perception of the level of care received by the patient, increasing the difficulties of accessing hospital services or being seen by a physician at home. Moreover, the reduction in the number of the territorial home visits to minimize the chances of viral spread has certainly led to a perceived decline in care. During the first months of 2020, in fact, the distribution of new daily cases of COVID-19 has mainly concerned the areas of North Sardinia [60]. Some difficulties in specialized care, due to the exacerbations of personnel shortage and the lack of long-term planning, have also affected the areas of Central and Southern Sardinia. In these two areas and in the Olbia-Tempio district, there was also a lack of psychological services dedicated to the patient, with the impossibility of having support during the pandemic period. Psychological support is also essential right after the communication of the diagnosis, and for the adaptive strategies to be adopted as the disease progresses [61,62,63,64].

Among all the caregivers, although moderate levels of care burden have been highlighted, a high level of perceived stress emerged. The caregiver often provided assistance to the family member for more than 50 h a week, in addition to his personal work activity. The continuous caring activities provided, in line with evidence, imply a possible reduction of the caregiver’s health state [22,64,65]. The negative correlation between the increased caregiver’s care burden and the QoL perceived by the ALS patient should be deepened, highlighting a potential obstacle to an adequate home care service.

The COVID-19 emergency period seems to have had a significant influence on the QoL of caregivers in Central Sardinia, with a moderate impact in the North and South. This fact could be explained by the caregiver’s perception of a decrease in the family member assistance level, the reduction of home care access, and the greater difficulty in obtaining medical check-ups. In addition, isolation with less possibility of having external support to the family unit, and the lack of psychological support, might have further exacerbated the care burden and stress perceived by the caregiver.

It is crucial to monitor regularly the care settings over time, with shared methodology and outcome indicators, to allow an objective comparison in the quality of care and its possible critical issues. To this end, it is also vital to complete the digitalization and integration of services, ensuring the traceability of data and the continuous monitoring activities.

The main limitation of this study is the small sample size. It is noteworthy that this study did not incorporate a control group, which may limit the external validity of the findings. Nevertheless, our results have provided crucial insights into the quality of life of individuals with ALS, which may inform existing care models and warrant further investigations. The methodology used with direct interviews, the good balance of the analysis sample and the evaluation by thematic domains, instead of individual items, makes our results reasonable, reducing confounders or bias. Another significant limitation of our study is the lack of a constructed Italian validation of the questionnaire used for patient interviews. Finally, the interviews might also have underestimated the real care and emotional load faced by the caregiver during the COVID-19 emergency period, due to the limitation of assessing the data retrospectively. A multicenter study might further clarify the role of the ALSSQOL-SF individual items on the subjects’ well-being.

## 5. Conclusions

The tools used for the face-to-face meeting with the ALS subjects and their caregivers have proven to be suitable for the aim of the study, allowing one to identify in an accurate, replicable, and uniform way the global features of the ALS patients’ QoL and the caregivers’ care burden. The direct mode of the interview, with the opportunity to have qualitative data for each local community, made it possible to deepen the reasons related to some critical issues, and limit information bias.

Results achieved through this study are vital to carefully define a proper care model that must consider the patients’ needs in their local setting and beyond their physical needs, supporting them and their family caregivers also in psychological and social well-being. 

## Figures and Tables

**Table 1:** Characteristics of the sample of subjects with ALS in the Sardinian Region (n = 66).

ALS Patients		North n, %	Center n, %	South n, %	Total n, %
Sex	F	12 (44.4)	2 (11.8)	8 (36.4)	22 (33.3)
	M	15 (55.6)	15 (88.2)	14 (63.6)	44 (66.7)
Age range (years)	<40	1 (3.7)	0 (0)	0 (0)	1 (1.5)
	41–50	1 (3.7)	0 (0)	3 (13.6)	4 (6.1)
	51–60	7 (25.9)	6 (35.3)	7 (31.9)	20 (30.3)
	61–70	9 (33.4)	7 (41.2)	5 (22.7)	21 (31.8)
	71–80	5 (18.5)	3 (17.6)	5 (22.7)	13 (19.7)
	>80	4 (14.8)	1 (5.9)	2 (9.1)	7 (10.6)
CLIS/FTD	No	21 (77.8)	12 (70.6)	15 (68.2)	48 (72.7)
	Yes	6 (22.2)	5 (29.4)	7 (31.8)	18 (27.3)
Tracheostomy	No	12 (44.4)	3 (17.6)	8 (36.4)	23 (34.8)
	Yes	15 (55.6)	14 (82.4)	14 (63.6)	43 (65.2)
Gastrostomy	No	9 (33.3)	3 (17.6)	5 (22.7)	17 (25.8)
	Yes	18 (66.7)	14 (82.4)	17 (77.3)	49 (74.2)

**Table 2:** ALSSQOL-SF questionnaire results for the sample of patients not showing completely locked in state (CLIS) or frontotemporal dementia (FTD).

Items ALSSQOL-SF ^1^	Median	Percentile 25	Percentile 75	Mode
Global quality of life, 30 days before	5	4	8	5
1. Pain	4	0	6	0
2. Fatigue	8	5	9	8
3. Excessive saliva	5	0	8	0
4. Communication issues	3	0	7	0
5. Strength and ability to move	9	8	10	10
6. Sleep disorders	4	1	8	0
7. Feeling physically terrible	5	4	8	4
8. Caring and needs-sensitive world	6	4	9	10
9. Feeling supported	9	6	10	10
10. Depression	4	1	8	0
11. Satisfying relationships with the closest people	9	7	10	10
12. Religion source of strength or comfort	7	2	10	10
13. Feeling religious or spiritual	7	4	9	10
14. Feeling hopeless	5	1	8	0
15. Sadness	5	3	7	0 ^a^
16. Enjoying the beauty of the surroundings	9	7	10	10
17. Desire to communicate their emotions	7	3	10	10
18. Express their emotions	5	3	8	3 ^a^
19. Desire for physical contact	7	4	9	10
20. Sufficient physical contact	8	4	10	10
21. Commitment to others in the community	5	3	9	10
22. Appreciation of time spent with other people	8	5	10	10
**COVID-19 emergency period**				
23. Worsening of the quality of life	5	0	8	0
24. Loneliness	4	0	7	0
25. Proper medical care	7	2	8	7
26. Adequate psychological support	3	0	8	0
27. Lack of physical contact	5	2	8	0
28. Inability to perform some activities	6	1	8	0
29. Difficult relationship with people during care	1	0	5	0
30. Greater difficulty communicating	2	0	5	0

^1^ For the items 1–30, the value 0 represented the expression “strongly disagree” with the statement, 10 the expression “strongly agree”. Values 1, 2, and 3 were expressed “disagreeing” with the statement, 4 “partially disagreeing”, 5 “neither agree nor disagree”, 6 “partially agreeing”, 7, 8, and 9 “agreeing” with the statement. Note, “a” points out that there are multiple modal values; the smallest value is displayed.

**Table 3:** Ordinal logistic regression model assessing the influence of age, gender, negative emotions, interactions with people and the environment, physical and emotional intimacy, spirituality, physical symptoms, and bulbar function on the perceived global QoL among ALS patients.

Ordinal Regression Number of obs = 47					
LR chi^2^(8) = 32.62					
Prob > chi^2^ = 0.0001					
Loglikelihood = −83.		Pseudo R^2^ = 0.1641			
QOL GL	Odds Ratio	z	*p* > |z|	(95% Conf. Interval)	
Age	0.9394915	−0.23	0.815	0.5562592	1.58675
Sex	1.559553	0.73	0.468	0.4698767	5.176261
Negative emotions	0.7020899	−2.77	0.006	0.5464532	0.9020537
People/environment interactions	1.217894	1.05	0.295	0.8423722	1.76082
Physical/emotional intimacy	1.559291	2.56	0.010	1.110363	2.189726
Spirituality	0.9818377	−0.19	0.846	0.8162948	1.180952
Physical Symptoms	0.705342	−1.79	0.074	0.4811217	1.034057
Bulbar function	1.009111	0.08	0.934	0.8154303	1.248796

**Table 4:** General characteristics of the sample of caregivers in Northern, Central, and Southern Sardinia.

Caregivers		North	Center	South	Total
Age (mean, SD)		56 ± 13	55 ± 14	60 ± 13	57 ± 13
Sex	F	22	17	18	57
	M	8	3	8	19
Time interval Starting Assistance-Diagnosis (days)	Median	−31	0	0	0
	IQR	−304–61	0–199	−123–31	−151–92
Weekly Assistance (hours)	Mode	>50	>50	>50	>50

**Table 5:** Results of the Zarit Burden Interview questionnaire for the sample of caregivers (including caregivers of subjects with locked-in status and frontotemporal dementia).

Items Zarit Burden Interview (ZBI) ^1^	Median	Percentile 25	Percentile 75	Mode
1. Patient’s requests to family versus his/her real need	1	0	5	0
2. Time for yourself is missing	8	4	10	10
3. Stress in caring for the family member and coping with other responsibilities	8	3	10	10
4. Embarrassment for the behavior of the family member	0	0	0	0
5. Feeling angry during patient’s care	0	0	3	0
6. Negative influence of the family member in the relationship with family and friends	0	0	2	0
7. Fear for the future of the family member	10	7	10	10
8. Perception of the family member’s dependence on their role as caregivers	10	8	10	10
9. Emotional distress during care	0	0	4	0
10. Perceived deterioration of health status in relation to one’s role as a caregiver	6	1	9	0 ^a^
11. Not having the desired private life	5	1	9	0
12. Worsening of social life for the care activity carried out	7	2	10	10
13. Discomfort in inviting friends at home	0	0	0	0
14. Perception of the need perceived by the family member with regard to their role as caregiver	8	2	10	10
15. Perception of economic difficulty	6	2	10	10
16. No longer being able to take care of the family member	2	0	8	0
17. Having lost control of your life from the moment your family member fell ill	5	0	9	0
18. Entrust the care of the family member to someone else	0	0	1	0
19. Insecurity about what to do for the family member	2	0	7	0
20. Perception of the need to do more for the family member	4	0	7	0
21. Perception of the need to do better in the care of the family member	3	0	6	0
22. Perception of being overloaded in the care of the family member	5	1	9	0 ^a^
**COVID-19 emergency period**				
23. Worsening QoL	5	0	10	0
24. Perceived loneliness	3	0	9	0
25. Proper medical care to the family member	6	2	9	10
26. Adequate psychological support	0	0	5	0
27. Inability to perform some activities	6	1	9	0 ^a^
28. Difficult relationship with the family member	0	0	6	0
29. Support they felt they received	4	0	8	0

^1^ For items 1–29, the value 0 represents the expression “strongly disagree” with respect to the statement, and 10 the expression “strongly agree”. The values of scale 1, 2, and 3 were expressed as “disagreeing” with the statement, 4 “partially disagreeing”, 5 “neither agree nor disagreeing”, 6 “partially agreeing”, 7, 8, and 9 “agreeing” with the statement. Note “a” indicates that there are multiple modal values, the smallest value is displayed.

## Data Availability

The data presented in this study are available on reasonable request from the corresponding author.

## Supplementary Materials

The following supporting information can be downloaded at: https://www.mdpi.com/article/10.3390/healthcare11111641/s1, Section S1, Modified version of ALSSQOL-SF questionnaire (original language); Section S2, Modified version of Zarit burden Interview (original language).
