# Supplementary material for: Quality of Life in Amyotrophic Lateral Sclerosis Patients and Care Burden of Caregivers in Sardinia during COVID-19 Pandemic
Source: Healthcare (Basel). 2023 Jun 3;11(11):1641. doi: 10.3390/healthcare11111641 (PMC10252463; doi:10.3390/healthcare11111641)
Supplement: Supplementary file 1 [file healthcare-11-01641-s001.zip › healthcare-2109738-supplementary.pdf]

Section S1 - Questionario sulla qualità della vita specifico per la Sclerosi Laterale Amiotrofica-Forma breve (ALSSQOL-SF) adattato per periodo dell'emergenza COVID-19

Età ≤40 □ 41 -50 □ 51-60 □ 61-70 □ 71-80 □ > 80 □

Sesso M □ F□

Indicazioni:

Le domande del questionario iniziano con una dichiarazione seguita da due risposte opposte. I numeri si estendono da una risposta estrema al suo contrario. Indichi il numero compreso tra 0 e 10 che rappresenta di più il suo stato nell'ultimo mese. Non ci sono risposte giuste o sbagliate. Le risposte completamente oneste saranno molto utili. È possibile non rispondere alla domanda se dovesse ritenerla troppo personale.

Le chiedo di valutare la sua qualità di vita nell'ultimo mese (30 giorni):

		Pessima										Ottima
	Considerando tutte le parti della mia vita – fisica, emotiva, sociale, spirituale e finanziaria – nell'ultimo mese, la qualità della mia vita è stata:	0	1	2	3	4	5	6	7	8	9	10

Le chiedo di indicare per le seguenti affermazioni quanto è d'accordo o in disaccordo con ognuna di esse.

		Fortemente in disaccordo										Fortemente d'accordo
1	Ho provato dolore	0	1	2	3	4	5	6	7	8	9	10
2	Ho provato stanchezza	0	1	2	3	4	5	6	7	8	9	10
3	Ho avuto un'eccessiva salivazione	0	1	2	3	4	5	6	7	8	9	10

4	Ho avuto difficoltà a comunicare	0	1	2	3	4	5	6	7	8	9	10
5	Ho avuto problemi con la forza e la capacità di spostarmi	0	1	2	3	4	5	6	7	8	9	10
6	Ho avuto problemi a dormire	0	1	2	3	4	5	6	7	8	9	10
7	Sono stato in un pessimo stato fisico	0	1	2	3	4	5	6	7	8	9	10
8	Il mondo è stato attento e sensibile ai miei bisogni	0	1	2	3	4	5	6	7	8	9	10
9	Mi sono sentito sostenuto	0	1	2	3	4	5	6	7	8	9	10
10	Mi sono sentito depresso	0	1	2	3	4	5	6	7	8	9	10
11	Le relazioni con le persone a me più vicine sono state soddisfacenti	0	1	2	3	4	5	6	7	8	9	10
12	La mia religione è stata per me una fonte di forza o conforto	0	1	2	3	4	5	6	7	8	9	10
13	Mi considero religioso o spirituale	0	1	2	3	4	5	6	7	8	9	10
14	Mi sono sentito senza speranze	0	1	2	3	4	5	6	7	8	9	10
15	Mi sono sentito triste	0	1	2	3	4	5	6	7	8	9	10
16	Ho apprezzato la bellezza di ciò che mi circonda	0	1	2	3	4	5	6	7	8	9	10
17	Ho avuto un forte desiderio di comunicare le mie emozioni	0	1	2	3	4	5	6	7	8	9	10
18	Ho comunicato le mie emozioni agli altri	0	1	2	3	4	5	6	7	8	9	10
19	Ho avuto un forte desiderio di contatto fisico	0	1	2	3	4	5	6	7	8	9	10
20	Ho avuto un contatto fisico con gli altri	0	1	2	3	4	5	6	7	8	9	10
21	Mi sono impegnato per altre persone nella mia comunità	0	1	2	3	4	5	6	7	8	9	10
22	Ho apprezzato il tempo trascorso con altre	0	1	2	3	4	5	6	7	8	9	10

Vorremmo infine porle alcune brevi domande relative al periodo dell'emergenza COVID-19

		Fortemente in disaccordo									Fortemente d'accordo
23	L'emergenza COVID-19 ha peggiorato la mia qualità di vita	0	1	2	3	4	5	6	7	8	9	10
24	Mi sono sentito solo rispetto a prima dell'emergenza	0	1	2	3	4	5	6	7	8	9	10
25	Ho avuto cure adeguate durante l'emergenza	0	1	2	3	4	5	6	7	8	9	10
26	Ho avuto supporto psicologico adeguato	0	1	2	3	4	5	6	7	8	9	10
27	Ho avvertito di più la mancanza di un contatto fisico	0	1	2	3	4	5	6	7	8	9	10
28	Non ho potuto svolgere alcune attività che per me erano importanti	0	1	2	3	4	5	6	7	8	9	10
29	Il rapporto con le persone che mi assistono è stato più difficile	0	1	2	3	4	5	6	7	8	9	10
30	Ho avuto maggiori difficoltà a comunicare	0	1	2	3	4	5	6	7	8	9	10

Section S2 - Zarit Burden Interview (ZBI) adattato per periodo dell'emergenza COVID-19

Età _____

Sesso M □ F □

Data diagnosi familiare (mese e anno) _____________

La persona che assiste ha la tracheostomia Sì □ No □ Da quanti mesi? _________

La persona che assiste ha la gastrostomia (PEG) Sì □ No □ Da quanti mesi? _________

Data di inizio assistenza al familiare (mese e anno) _____________

Ore assistenza settimanale <10 □ 10-20 □ 21-30 □ 31-40 □ 41-50 □ > 50 □

Le chiedo di indicare per le seguenti affermazioni quanto è d'accordo o in disaccordo con ognuna di esse

		Fortemente in disaccordo										Fortemente d'accordo
1	Ritiene che il suo familiare le chieda un aiuto maggiore rispetto a quello di cui ha bisogno?	0	1	2	3	4	5	6	7	8	9	10
2	Ritiene di non avere abbastanza tempo per sé stesso a causa del tempo impegnato nella cura del suo familiare?	0	1	2	3	4	5	6	7	8	9	10
3	Si sente stressato dall'avere cura del suo familiare e dal cercare di fare fronte alle altre responsabilità, familiari e di lavoro?	0	1	2	3	4	5	6	7	8	9	10
4	Si sente in imbarazzo per il comportamento del suo familiare?	0	1	2	3	4	5	6	7	8	9	10

5	Si sente arrabbiato quando è con il suo familiare?	0	1	2	3	4	5	6	7	8	9	10
6	Ritiene che il suo familiare influisca attualmente in maniera negativa sul suo rapporto con gli altri membri della famiglia e con gli amici?	0	1	2	3	4	5	6	7	8	9	10
7	Teme ciò che il futuro riserva al suo familiare?	0	1	2	3	4	5	6	7	8	9	10
8	Sente che il suo familiare è dipendente da lei?	0	1	2	3	4	5	6	7	8	9	10
9	Si sente teso quando assiste il suo familiare?	0	1	2	3	4	5	6	7	8	9	10
10	Ritiene che la sua salute ne abbia risentito a causa del prendersi cura del suo familiare?	0	1	2	3	4	5	6	7	8	9	10
11	Ritiene di non avere la vita privata che vorrebbe a causa dell'assistenza al suo familiare?	0	1	2	3	4	5	6	7	8	9	10
12	Ritiene che la sua vita sociale abbia risentito dal prendersi cura del suo familiare?	0	1	2	3	4	5	6	7	8	9	10
13	Si sente a disagio ad invitare a casa gli amici a causa del suo familiare?	0	1	2	3	4	5	6	7	8	9	10
14	Ritiene che il suo familiare si aspetti che lei si prenda cura di lui come se fosse l'unica persona da cui dipende?	0	1	2	3	4	5	6	7	8	9	10
15	Sente di non aver abbastanza denaro per prendersi cura del suo familiare in aggiunta alle sue spese personali?	0	1	2	3	4	5	6	7	8	9	10

16	Pensa di non farcela a prendersi cura del suo familiare ancora per molto tempo?	0	1	2	3	4	5	6	7	8	9	10
17	Pensa di non avere più il controllo della sua vita dal momento in cui il suo familiare si è ammalato?	0	1	2	3	4	5	6	7	8	9	10
18	Desidererebbe affidare la cura del suo familiare a qualcun altro?	0	1	2	3	4	5	6	7	8	9	10
19	Si sente insicuro su cosa fare per il suo familiare?	0	1	2	3	4	5	6	7	8	9	10
20	Sente che dovrebbe fare di più per il suo familiare?	0	1	2	3	4	5	6	7	8	9	10
21	Crede che potrebbe fare di meglio nella cura del suo familiare?	0	1	2	3	4	5	6	7	8	9	10
22	Nel complesso, si sente sovraccaricato dall'avere cura del suo familiare?	0	1	2	3	4	5	6	7	8	9	10

Vorremmo infine porle alcune brevi domande relative al periodo dell'emergenza COVID-19

		Fortemente in disaccordo										Fortemente d'accordo
23	Sente che l'emergenza COVID-19 abbia peggiorato la sua qualità di vita?	0	1	2	3	4	5	6	7	8	9	10
24	Si è sentito solo rispetto a prima dell'emergenza?	0	1	2	3	4	5	6	7	8	9	10
25	Ritiene che il familiare che assiste abbia avuto cure adeguate durante l'emergenza?	0	1	2	3	4	5	6	7	8	9	10

26	Ha avuto supporto psicologico adeguato durante l'emergenza?	0	1	2	3	4	5	6	7	8	9	10
27	Sente di non aver potuto svolgere alcune attività che per lei erano importanti?	0	1	2	3	4	5	6	7	8	9	10
28	Il rapporto con la persona che assiste è stato più difficile?	0	1	2	3	4	5	6	7	8	9	10
29	Si è sentito sostenuto?	0	1	2	3	4	5	6	7	8	9	10
